# Zombie Cruise Ship Virtual Escape Room for POCUS Pulmonary: Scan Your Way Out

**DOI:** 10.21980/J8RM0M

**Published:** 2022-07-15

**Authors:** Heesun Choi, Alisa Wray, Jonathan Smart

**Affiliations:** * University of California Irvine, Department of Emergency Medicine, Orange, CA; ^ Kingman Regional Medical Center, Department of Emergency Medicine, Kingman, AZ

## Abstract

**Audience:**

Targeted audience could be learners in medical field with basic knowledge of point-of-care ultrasound (POCUS), pulmonary and emergency medicine for example, medical students, emergency medicine residents (1^st^–3^rd^ year), emergency physicians at all level of trainings, or emergency medicine physician’s assistants.

**Introduction:**

Point-of-care ultrasound (POCUS) is rapidly becoming an essential part of emergency medicine and patient care .^1^,^2^ POCUS can provide more detailed clinical information when used in conjunction with a physical examination, overall aiding clinicians’ decision-making capacity.^3^ POCUS also proves a cost-effective tool in reducing the number of advanced imaging studies ordered and unnecessary patient radiation exposure.^3^,^4^ Performing POCUS has also proved beneficial for patient satisfaction because it increases the amount of face-to-face time spent with the patient while also providing live imaging interpretation during the emergency department visits .^3^,^5^,^6^ POCUS-Pulmonary can also create a safer environment for both medical staff and patients during the COVID-19 pandemic.^6^ Performing POCUS-Pulmonary on suspected COVID-19 patients can limit the number of patients receiving thoracic CT studies to confirm COVID-19 related pneumonia.^6^,^7^ Performing POCUS-Pulmonary reduces the number of patients transferred between the radiology department and the emergency department, significantly reduces overall possible COVID-19 exposures, and reduces equipment cleaning time.^6^ Given the overall reduction of advanced imaging studies ordered, CT scanners would be more readily available for critical care patients, such as trauma or other hemodynamic instability.^6^ Emergency providers practicing in rural areas with limited resources may benefit from the use of POCUS -Pulmonary, facilitating better patient care at decreased exposure-rate, cleaning cost, and overall increase in patient satisfaction given more bedside patient-provider communication.^6^–^8^ POCUS-Pulmonary is a crucial clinical skill for emergency medicine providers everywhere.^6^,^8^ Clinicians should be able to perform POCUS-Pulmonary, interpret image findings, and develop a treatment plan promptly.^9^

**Educational Objectives:**

By the end of performing the Zombie Cruise Ship Virtual Escape Room, learners will be able to: 1) recognize sonographic signs of A-line, B-line, Barcode sign, Bat sign, Seashore Sign, Plankton sign, Jellyfish Sign, Lung point, lung lockets, and Lung pulse; 2) differentiate sonographic findings of pneumothorax, hemothorax, pneumonia, COVID 19 pneumonia, pulmonary edema, and pleural effusion from normal lung findings; 3) distinguish pneumonia from atelectasis by recognizing dynamic air bronchogram; and 4) recognize indications for performing POCUS pulmonary such as dyspnea, blunt trauma, fall, cough and/or heart failure.

**Educational Methods:**

This group-based learning program was designed for use in virtual meetings, lectures, and in small-group learning activities, such as didactic and EM conferences. A Google form was used to create a virtual escape room for learners in which they had to take quizzes to advance to the next level. Learners may enact teamwork through discussion and group effort, or respond individually to ultrasound pulmonary questions.

**Research Methods:**

Learners will take pre and post-test assessment to compare the learners POCUS-Pulmonary knowledge before and after small group, virtual escape room learning. All participants in the virtual escape room game are given a pre and post-test assessment comprised of seventeen total questions: two questions asking the participant's training level, and fifteen POCUS-Pulmonary questions. Pre and Post-test questions are identical; however, the participants' answers to the pre-test assessment are not revealed to them on completion. Instead, participants receive a letter grade on completing the pre-test assessment. Participants complete the pre and post-test assessments over fifteen minutes allotted before and after the virtual escape room. Upon completing the post-test assessment, a letter grade and the correct answers were given to the participants.

**Results:**

Twenty-four emergency medicine resident physicians (PGY 1–3)) participated in the Zombie Cruise Ship Escape Room pre-test, while a total of twenty-three resident physicians participated in the post-test assessment. The pre-test data showed an average of 10.33 points, compared to post-test data, which showed 11.91 points. There was an improvement of two points on the median score with a median pre-test score of 10 vs. the post-test median of 12.

**Discussion:**

The virtual zombie cruise ship experience proved a practical and useful tool in increasing overall participants' interest in POCUS pulmonary during the COVID-19 pandemic. Participants had higher retention after actively discussing and researching the most up-to-date clinical information during the virtual and inperson small group meetings. The game encouraged participants to make decisions quickly. This pace created a fun competition between participants who genuinely enjoyed the learning experience even during the COVID-19 pandemic via Zoom/Google Meet virtual conferences. By creating a virtual escape learning tool, learners can experience teamwork-based learning without concern for group size limitations during the pandemic.

**Topics:**

Sonographic findings of pneumothorax, hemothorax, pneumonia, COVID-19 pneumonia, pulmonary edema, pleural effusion, normal lung, A-line, lack of A-line, presence of B-line, Lung sliding, M mode, dynamic air bronchogram, lung rockets, Bar code Sign, Bat Sign, lung pulse, lung point, hepatization, Seashore Sign, Plankton Sign, Jellyfish Sign, and subpleural pulmonary consolidation

## USER GUIDE

**Table:** 

**List of Resources:**	
Abstract	1
User Guide	4
Appendix A: Scan Your Way Out Map – Syllabus for Instructors	8
Appendix B: Pre & Post Test Questions	9
Appendix C: Pre & Post Test Answers	13
Appendix D: Zombie Cruise Ship Google Form Link and PDF	18
Appendix E: Zombie Cruise Ship Questions & Answers	19
Appendix F: Zombie Cruise Ship References for images in the Google Forms Document	20
Appendix G: Zombie Cruise Ship Instructor Pearls	22

**Learner Audience:**

Medical Students, Interns, Junior Residents, Senior Residents, Physician’s Assistants, Fellows, Attending Physicians

**Time Required for Implementation:**

40 minutes

**Recommended Number of Learners per Instructor**:

5–10

**Topics:**

Sonographic findings of pneumothorax, hemothorax, pneumonia, COVID-19 pneumonia, pulmonary edema, pleural effusion, normal lung, A-line, lack of A-line, presence of B-line, lung sliding, M mode, dynamic air bronchogram, Lung rockets, Bar code Sign, Bat Sign, lung pulse, lung point, hepatization, Seashore Sign, Plankton Sign, Jellyfish Sign, and subpleural pulmonary consolidation.

**Objectives:**

By the end of the Zombie Cruise Ship Virtual Escape Room, learners will understand basic knowledge of POCUS pulmonary. After 45-minutes of virtual team-based small group activity, the learners will be able to:

1. Recognize sonographic signs of A-line, B-line, Barcode sign, Bat sign, Seashore Sign, Plankton sign, Jellyfish Sign, Lung point, lung lockets, and Lung pulse
2. Differentiate sonographic findings of pneumothorax, hemothorax, pneumonia, COVID 19 pneumonia, pulmonary edema, and pleural effusion from normal lung findings
3. Distinguish pneumonia from atelectasis by recognizing dynamic air bronchogram
4. Recognize indications for performing POCUS pulmonary such as dyspnea, blunt trauma, fall, cough and/or heart failure.

### Linked objectives and methods

The Zombie Cruise ship scenario's goal was to measure the efficacy of small group-based distance learning programs for POCUS Pulmonary during the COVID 19 pandemic. This virtual escape room was created to make the virtual learning experience fun and engaging during the COVID-19 pandemic and was motivated by a desire to decrease Zoom meeting and Google meeting fatigue. Students at all levels were isolated during the COVID-19 pandemic, at no small detriment to their mental and educational pursuits. This activity was intended to inspire distanced learners through online human interaction as if they could physically play games together.

All participants should take the pre-test prior to entering the Zombie Cruise Ship Virtual Escape Room. This pre-test was designed to measure the learners’ knowledge of POCUS pulmonary and to prepare for the virtual escape room questions. Learns will be able to recognize their lack of knowledge in POCUS pulmonary which will encourage learners to engage more in the session to fill in their knowledge gaps. After escaping the Zombie Cruise Ship, the learners should take the post-test. The pre- and post-test questions are the same questions. They are designed to measure how much learners have learned from participating in the virtual escape room.

Working through the Zombie Cruise Ship Escape Room allows the learners to answer questions and work through cases that will teach each of the objectives. They will identify the pulmonary ultrasound findings from short video clips within the virtual escape room (objective 1). Differentiate sonographic findings of pneumothorax, hemothorax, pneumonia, COVID 19 pneumonia, and pulmonary edema from normal lung findings as the participants decide to help virtual cruise ship guests who are in need of medical assistance either due to a chronic medical condition, acute illness and/or trauma (objective 2). Learners must recognize pathologies from the POCUS pulmonary video clips and choose appropriate medical treatment in each scenario (objective 3 and 4). The escape room is designed to use the portable ultrasound to evaluate each complaint from virtual cruise ship guests to be able to either provide medical treatment to save the guests or to escape from the zombies.

### Recommended pre-reading for facilitator

- Marini TJ, Rubens DJ, Zhao YT, Weis J, O’Connor TP, Novak WH, et al. Lung ultrasound: the essentials. Radiol Cardiothorac Imaging. 2021;3(2):e200564. doi:10.1148/ryct.2021200564
- Eric Abrams, MD FACEP. Lung Ultrasound in COVID-19. ACEP Emergency Ultrasound. https://www.acep.org/how-we-serve/sections/emergency-ultrasound/news/june-2020/lung-ultrasound-in-covid-19---acep-ultrasoundsonoguide-subcommittee/

### Learner responsible content (LRC)

- Marini TJ, Rubens DJ, Zhao YT, Weis J, O’Connor TP, Novak WH, et al. Lung ultrasound: the essentials. Radiol Cardiothorac Imaging. 2021;3(2):e200564. doi:10.1148/ryct.2021200564
- Eric Abrams, MD FACEP. Lung Ultrasound in COVID-19. ACEP Emergency Ultrasound. https://www.acep.org/how-weserve/sections/emergency-ultrasound/news/june-2020/lung-ultrasound-in-covid-19---acep-ultrasoundsonoguide-subcommittee/

### Small group application exercise (sGAE)

- Zombie Cruise Ship Google Form Address: https://forms.gle/ec1ErE6niPt2RDQPA

### Results and tips for successful implementation

The Zombie Cruise Ship scenario is best implemented through either a Zoom meeting or in physical small group meetings.

A small group of 5 to 10 learners in one Zoom meeting is ideal. The learners must turn their video on and be able to type answer choices. The learners will open the zombie cruise ship link (https://forms.gle/ec1ErE6niPt2RDQPA) on their computer. The instructor should pick a learner to read the question, ask participants to type their answers, and pick the most answered choice. The instructor should pick different learners to read the next question. The instructor should also let the learners discuss each other by letting them unmute and freely type on the chat. After the post-test, the instructor can review questions together and summarize learning points.

A small group of five-six learners can do a physical meeting. Each group will need one instructor to encourage every participant to finish on time. The instructor should not help with any questions while learners are solving the zombie escape room. Each group can have either one computer for each participant or one computer per group of learners. If the group has only one computer, learners can gather around the computer and solve the questions together. If the group has an individual computer, the learners can only move onto the next questions after everyone agrees with the best answer. It is encouraged to allow the learners to struggle on questions and for the instructor to provide elaboration after learners have selected the correct answer.

All participants in the virtual escape room game are given a pre and post-test assessment comprised of seventeen total questions: two questions asking the participant's training level, and fifteen POCUS-Pulmonary questions. Pre and Post-test questions are identical; however, the participants' answers to the pre-test assessment are not revealed to them on completion. Instead, participants receive a letter grade on completing the pre-test assessment. Participants complete the pre and post-test assessments over fifteen minutes allotted before and after the virtual escape room. Upon completing the post-test assessment, a letter grade and the correct answers were given to the participants.

Twenty-four emergency medicine resident physicians (PGY-1 through PGY-3) participated in the Zombie Cruise Ship Escape Room pre-test, while a total of twenty-three resident physicians participated in the post-test assessment. The pre-test data showed an average of 10.33 points, compared to post-test data, which showed 11.91 points. There was a two points improvement of the median score with a median pre-test score of 10 vs. the post-test median of 12.

Additionally, the virtual escape room was presented at two different medical schools for third and fourth-year students via Zoom conference meetings: University of California Irvine School of Medicine (UCISOM) and Touro University Nevada College of Osteopathic Medicine (TUNCOM). At UCISOM, approximately fifty students participated the zombie cruise ship escape room during the Clinical Foundation course via Zoom meeting with one instructor. At TUNCOM, approximately thirty students participated in an emergency medicine interest group conference via Zoom with one instructor. Students have given positive feedback and expressed a greater interest in emergency medicine ultrasound rotations after participating in the zombie cruise ship escape room.

During these sessions, we learned that participants stayed focused during the entire small group session. This encouraged participants to actively discuss their clinical decision making process based on the zombie cruise ship scenarios. One of the questions in the zombie cruise ship scenario had two correct answers. However, to encourage more discussion, the question did not indicate that there were two correct answers. The participants engaged more with the small group activity by using evidence-based information to convince each other why their answers were correct. Participants were eager to reference their textbooks or clinical journals to support their reasoning.

**Figure:**
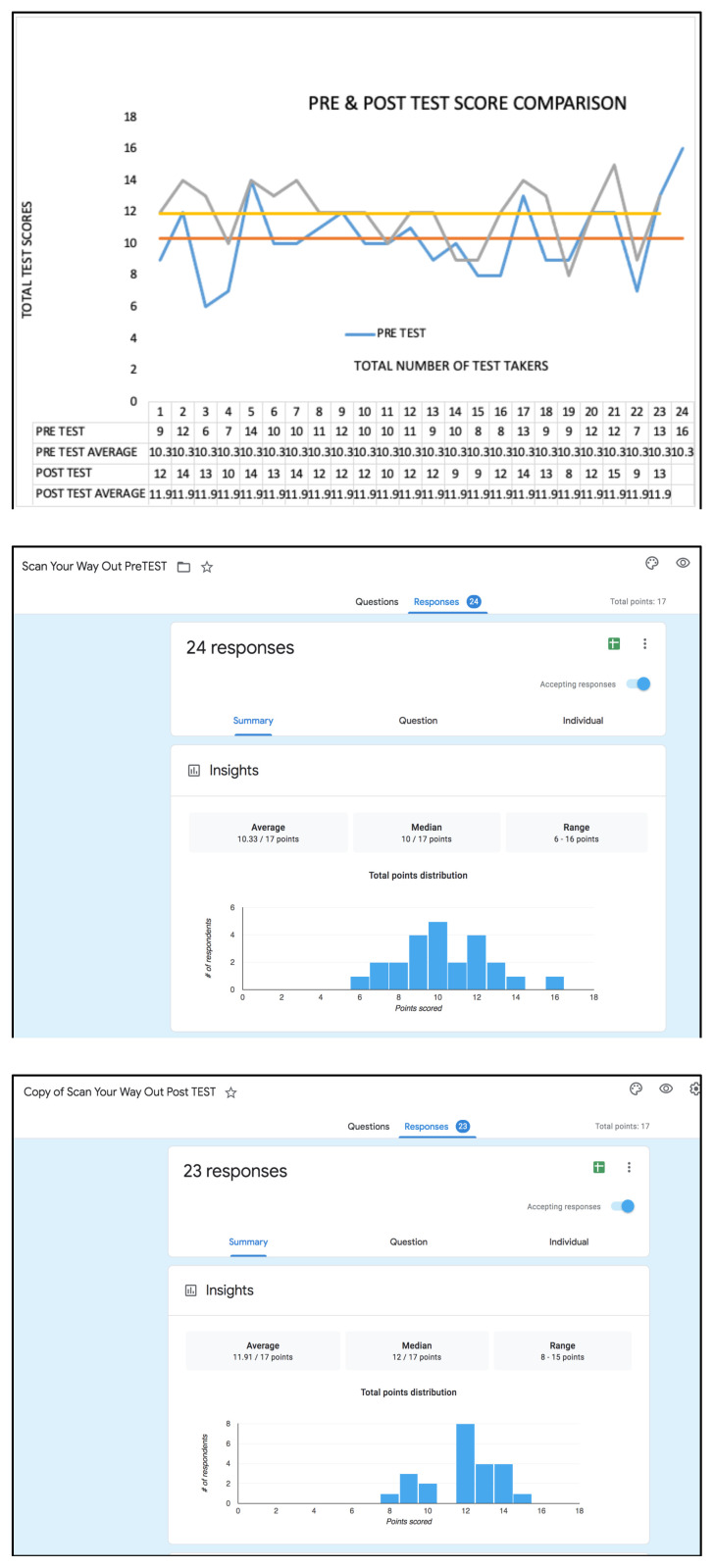


### Pearls

Please see attached document: Scan Your Way Out Map. Learners will be able to identify indications of POCUS pulmonary, recognize the sonographic signs of POCUS pulmonary, and differentiate different pulmonary pathologies by identifying unique sonographic findings of each pathology such as pneumonia, pneumothorax, hemothorax, pulmonary edema, pleural effusion, COVID 19 pneumonia, and atelectasis.

Specific skills are listed below:

1. Identify signs of absence of lung sliding
2. Use m-mode and are able to identify signs of pneumothorax
3. Recognize cardiac arrest using POCUS
4. Recognize pulmonary edema with increased B lines (More than 3 B lines)
5. Recognize the pulmonary edema due to heart failure on cardiac ultrasound by identifying decreased LVEF (left ventricle ejection fraction) and contractility
6. Describe an appropriate treatment for fluid overload
7. Diagnose pneumonia based on ultrasound findings: dynamic air bronchogram, hepatization, and loss of A lines
8. Distinguish difference between atelectasis vs. pneumonia findings on US
9. Identify hemothorax on a trauma patient
10. Identify pneumothorax on a trauma patient
11. Recognize ultrasound findings of COVID-19 pneumonia

## Figures and Tables

**Figure:**
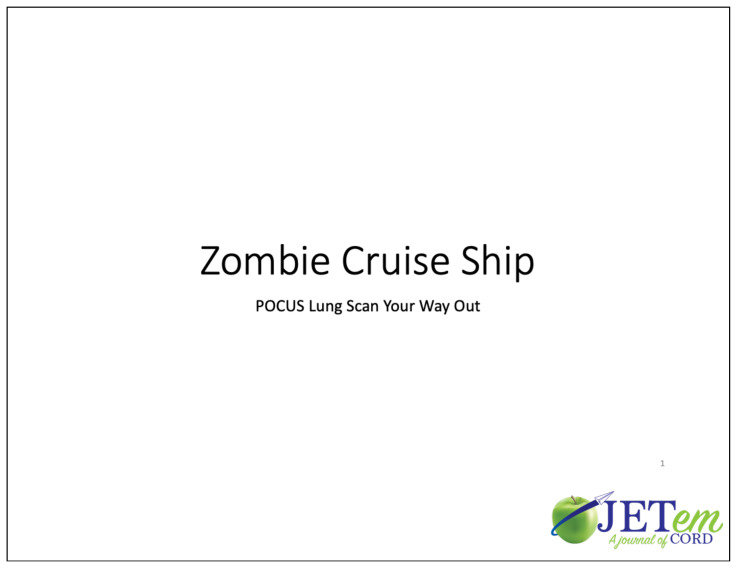
https://forms.gle/ec1ErE6niPt2RDQPA Please see associated PDF file

## SMALL GROUPS LEARNING MATERIALS

### Appendix A Scan Your Way Out Map—Syllabus for Instructors

PDF file.

### Appendix B Zombie Cruise Ship Pre & Post Test Questions

1. Which of the following probes is preferred to use to identify pneumothorax:
  - Curved Linear Probe
  - Linear Probe
2. While performing POCUS pulmonary, an absence of lung sliding could be diagnosed as a false positive pneumothorax in a patient with other pulmonary diseases. Which of the following conditions could present as an absence of lung sliding on POCUS pulmonary? (Multiple answers)
  - Pleural effusion (Positive B lines)
  - Chest tube (Negative B lines)
  - Chronic obstructive pulmonary disease (Negative B lines)
  - Consolidation with pleural adhesion (Positive B lines)
  - COVID 19 Pneumonia (Positive B lines)
3. What is the most likely pathognomonic finding in pneumonia?
  - Presence of 4 or more B lines
  - Loss of visceral parietal pleural interface
  - Presence of a “lung point”
  - Hepatization of the lung
  - Absence of A line
4. Please select normal lung findings on POCUS pulmonary (multiple answers)
  - A lines
  - B lines – more than 3
  - Lung point
  - Lung sliding
  - Absence of lung pulse
  - Air bronchograms
  - Seashore sign
  - Lung Hepatization
5. Which of the following POCUS findings has the highest specificity for diagnosing pneumothorax?
  - Positive A line, Absence B line, Positive Lung point
  - Absence A line, Absence B line, Positive Lung pulse
  - Absence A line, Positive B line, Positive Lung pulse
  - Positive A line, Positive B line, Positive Bat sign
6. What does a positive plankton sign on POCUS pulmonary most likely represent?
  - Pneumonia
  - Pneumothorax
  - Pulmonary edema
  - Pleural effusion
7. False positive lung points may be observed over pleural borders at the heart and diaphragm.
  - True
  - False
8. Which of following describes ‘lung pulse” finding on POCUS pulmonary?
  - Rhythmic movement of the pleura in synchrony with the cardiac rhythm
  - Highly specific ultrasound sign of pneumonia
  - An artifact of normal lung which is best visualized in hyperinflated lung
  - Hyper-echoic foci that may move with respirations within the lung parenchyma
9. Which of the following acronyms are most likely associated with the “RUSH Protocol?”
  - Pump, Tank, Pipes
  - Pump, Tire, Pipes
  - Scan, Tank, Probe
  - Pump, Wire, Pipes
10. What does the blue line represent in the POCUS pulmonary image?
  - The posterior chest wall
  - The rib pleura interface
  - The visceral parietal pleura interface
  - Pulmonary reverberation
11. Presence of lung point on POCUS pulmonary always rules out pneumothorax as a possible diagnosis.
  - Ture
  - False
12. Findings of dynamic air bronchograms on POCUS pulmonary favor diagnosis of atelectasis rather than pneumonia.
  - True
  - False
13. Which of the following describes lung rockets sign in POCUS pulmonary?
  - Consolidated pneumonia
  - Accumulation of fluid in the pulmonary interstitial space
  - Hyperechoic fluid collection within the lung parenchyma, loculated appearance
  - Rhythmic movement of the pleura in synchrony with the cardiac rhythm
14. Positive “lung worm sign” is seen on POCUS pulmonary when air is stuck within obstructed bronchi. This is highly specific for
  - Pneumothorax
  - Pleural effusion
  - COPD
  - Pneumonia
  - Hemothorax
  - COVID 19 pneumonia
15. Which of the following most likely describes “lung point” on POCUS pulmonary?
  - The boundary between normal lung and pneumonia
  - The boundary between normal lung and pneumothorax
  - The boundary between normal lung and hemothorax
  - The boundary between normal lung and abdominal cavity
  - The boundary between normal lung and cardiac cavity

### Appendix C Zombie Cruise Ship Pre & Post Test Answers

1. Which of the following probes is preferred to use to identify pneumothorax:
  - Curved Linear Probe
  - **Linear Probe**
    **Explanation:**
    Based on image quality, the linear array transducer might be preferred for lung ultrasound (pre hospital) for pneumothorax. ^(1)^
    A straight linear array high frequency probe (5–13 MHz) may be most helpful in analyzing superficial structures such as the pleural line and providing better resolution. ^(2)^
    **References**
    1. Ketelaars R. Which ultrasound transducer type is best for diagnosing pneumothorax? *Crit Ultrasound J*. 2018; 10:27.
    2. Husain L. Sonographic diagnosis of pneumothorax. *J Emerg Trauma Shock*. 2012 Jan–Mar; 5(1): 76–81.
2. While performing POCUS pulmonary, an absence of lung sliding could be diagnosed as a false positive pneumothorax in a patient with other pulmonary diseases. Which of the following conditions could present as an absence of lung sliding on POCUS pulmonary? (Multiple answers)
  - Pleural effusion (Positive B lines)
  - **Chest tube (Negative B lines)**
  - **Chronic obstructive pulmonary disease (Negative B lines)**
  - Consolidation with pleural adhesion (Positive B lines)
  - COVID 19 Pneumonia (Positive B lines)
    **Reference**
    1. Mallow C. Risk Factors for Loss of Lung Sliding in a Medical Intensive Care Population with Acute Respiratory Failure. *J Bronchology Interv Pulmonol*. 2019 Apr: 26(2) 102–107.
    2. Cirilli A. Ultrasound for Detection of Pneumothorax. REBELEM. At: https://rebelem.com/ultrasound-detection-pneumothorax/
3. What is the most likely pathognomonic finding in pneumonia?
  - Presence of 4 or more B lines
  - Loss of visceral parietal pleural interface
  - Presence of a “lung point”
  - **Hepatization of the lung**
  - Absence of A line
4. Please select normal lung findings on POCUS pulmonary (multiple answers)
  - **A lines**
  - B lines – more than 3
  - Lung point
  - **Lung sliding**
  - Absence of lung pulse
  - Air bronchograms
  - **Seashore sign**
  - Lung Hepatization
5. Which of the following POCUS findings has the highest specificity for diagnosing pneumothorax?
  - **Positive A line, Absence B line, Positive Lung point**
  - Absence A line, Absence B line, Positive Lung pulse
  - Absence A line, Positive B line, Positive Lung pulse
  - Positive A line, Positive B line, Positive Bat sign
6. What does a positive plankton sign on POCUS pulmonary most likely represent?
  - Pneumonia
  - Pneumothorax
  - Pulmonary edema
  - **Pleural effusion**
7. False positive lung points may be observed over pleural borders at the heart and diaphragm.
  - **True**
  - False
8. Which of following describes ‘lung pulse” finding on POCUS pulmonary?
  - **Rhythmic movement of the pleura in synchrony with the cardiac rhythm**
  - Highly specific ultrasound sign of pneumonia
  - An artifact of normal lung which is best visualized in hyperinflated lung
  - Hyper-echoic foci that may move with respirations within the lung parenchyma
9. Which of the following acronyms are most likely associated with the “RUSH Protocol?”
  - **Pump, Tank, Pipes**
  - Pump, Tire, Pipes
  - Scan, Tank, Probe
  - Pump, Wire, Pipes
10. What does the blue line represent in the POCUS pulmonary image?
  - The posterior chest wall
  - The rib pleura interface
  - **The visceral parietal pleura interface**
  - Pulmonary reverberation
11. Presence of lung point on POCUS pulmonary always rules out pneumothorax as a possible diagnosis.
  - Ture
  - **False**
12. Findings of dynamic air bronchograms on POCUS pulmonary favor diagnosis of atelectasis rather than pneumonia.
  - True
  - **False**
13. Which of the following describes lung rockets sign in POCUS pulmonary?
  - Consolidated pneumonia
  - **Accumulation of fluid in the pulmonary interstitial space**
  - Hyperechoic fluid collection within the lung parenchyma, loculated appearance
  - Rhythmic movement of the pleura in synchrony with the cardiac rhythm
14. Positive “lung worm sign” is seen on POCUS pulmonary when air is stuck within obstructed bronchi. This is highly specific for
  - Pneumothorax
  - **Pleural effusion**
  - COPD
  - Pneumonia
  - Hemothorax
  - COVID 19 pneumonia
15. Which of the following most likely describes “lung point” on POCUS pulmonary?
  - The boundary between normal lung and pneumonia
  - **The boundary between normal lung and pneumothorax**
  - The boundary between normal lung and hemothorax
  - The boundary between normal lung and abdominal cavity
  - The boundary between normal lung and cardiac cavity

### Appendix E Zombie Cruise Ship Questions & Answers

Please see associated PDF file

### Appendix F Zombie Cruise Ship References for Images in the Google Dorms Document

1. All the ultrasound video clips & images are from Dr. J. Christian Fox, MD. UC Irvine Department of Emergency Medicine
2. Google Form Section 2: 5: Best Practices of crowd control on ship by Trashcans Unlimited on 28^th^ Jan 2019. In: https://trashcansunlimited.com/blog/5-best-practices-of-crowd-control-on-ships/
3. Google Form Section 5 & 6: James Heilman, MD. An example of a person in Pinel restraints. In: https://en.wikipedia.org/wiki/Medical_restraint#/media/File:PinelRestaint.jpg
4. Google Form Section 5 & 6: J. Christian Fox, MD. Ultrasound Physics and Instrumentation. In: https://www.youtube.com/watch?v=531t0deTQwg
5. Google Form Section 11 and 12: Zombie CloseUp. In: https://www.flickr.com/photos/74568665@N03/10742806756
6. Google Form Section 15: Hyperlight. Dancing at a foam party; the blue object on the ceiling is a foam generator. In: https://en.wikipedia.org/wiki/Foam_party#/media/File:HLR-FOAM-PARTY-02.JPG. Creative Comment Attribution 2.5
7. Google Form Section 15: In: https://lh4.googleusercontent.com/gAXrYyc6i7o7DYkf7VqIwmyPMnp5tjgZ9Hz2UULAk6ConExP99kPKuTonyq3kYrGqB-jwnxIj7piKjqJPC7oTy7ixPK-R0rxK9SctpHtI4V5OzwzxcArHt7F6tM2dOwkTYY-2f6WPXN-D58=s0
8. Google Form Section 16: U.S. Air Force photo by Sgt. Ashley Taylor. Combined Joint Task Force--Horn of Africa. In: https://www.dvidshub.net/image/6193569/medlog-distributes-ppe-outstations-preventive-measures-covid-19
9. Google Form Section 16: Pet First Aid Kits. In: https://www.whiskerdocs.com/articles/pet-first-aid-kits
10. Google Form Section 16: U.S. Air Force photo illustration/Airman 1^st^ Class Daniel Phelps. Zombie attack. In: https://www.shaw.af.mil/News/Photos/igphoto/2000236415/
11. Google Form Section 18: CDC, Person in PPE. In: https://www.pexels.com/photo/person-in-ppe-3992939/
12. Google Form Section 26 and 27: James Heilman, MD. Pitting edema during and after the application of pressure to the skin. Creative Commons Attribution--Share Alike 3.0 Unported license. In: https://en.wikipedia.org/wiki/File:Combinpedal.jpg
13. Google Form Section 30: In: https://lh3.googleusercontent.com/55GzWcIxpAasaUwBnBm7v3RgeCZaNsbO7Wur_lfnkInkSelRjIkiyQFt4_TBSeTU3xw3-eRfHnQZXfsAiFZ6pgUrptvvb7eFse1yDVBJ-RG_9bNqgBzutcGUpDqraFk2HnuVhJWgghrzQ7I=s0
14. Google Form Section 31: Eden, Janine, and Jim. Old Woman in Bed Sculpture by Ron Mueck. In: https://www.flickr.com/photos/edenpictures/43521819642
15. Google Form Section 32 and 33: U.S. Air Force Photo by Staff Sgt. Michael C Zimmerman/Released. Service members give the gift of health in Peru. In: https://www.jble.af.mil/News/Photos/igphoto/2000133849/
16. Google Form Section 44: Sammy Williams on Unsplash. In: http://purvabrown.com/overcoming-challenges-fitness-screwing-up/
17. Google Form Section 52 and 53: Photo via Pixabay. In: https://www.shmadrid.com/blog/en/wp-content/uploads/2019/02/venice-2092601_1920.jpg
18. Google Form Section 52 and 53: Andrew Magill. Needle. In: https://commons.wikimedia.org/wiki/Category:Hypodermic_needles#/media/File:Syringe_Needle.jpg
19. Google Form Section 54 and 55: Photo by Pixabay. In: https://www.pikist.com/free-photo-xrscb
20. Google Form Section 56 and 57: Photo by Pixabay. In: https://pixabay.com/photos/captain-ferry-passenger-ship-2408590/
21. Google Form Section 60: Geo Swan. In: https://commons.wikimedia.org/wiki/File:Equinox_-_Lifeboat_Used_as_Tender.jpg
22. Google Form Section 60: Hobo Ringmaster. Hell yea. In: https://youtu.be/13YbqhCAJ7A
23. Google Form Section 61: Photo by Pixabay. In: https://www.pikist.com/free-photo-snjcw
24. Google Form Section 61: Starwars. In: https://tenor.com/view/starwors10-anoinevideo-gif-21159649

### Appendix G Zombie Cruise Ship Instructor Pearls

- **Visceral-parietal pleural interface**
  ○ Under normal conditions, the parietal and visceral pleura are visualized as a single hyperechoic line known as the pleural line.
- **A-lines**
  ○ The space in between each A line corresponds to the same distance between the skin surface and the parietal pleura.
  ○ A lines will be present in a patient with pneumothorax, but B lines will not.
  ○ A line with no lung sliding – sensitivity and specificity for an occult pneumothorax is as high as 95% and 94%.
- **B line**
  ○ Hyperechoic vertical lines that extend from the pleura to the edge of the screen without fading.
  ○ Comet-tail artifacts move synchronously with lung sliding and respiratory movement.
  ○ Three or more B lines noted in at least two bilateral lung fields is considered pathologic and concerning for pulmonary edema
  ○ B lines are the ultrasound equivalent of the Kerley B lines found on chest x-ray
- **Sonographic Signs of Pneumothorax**
  ○ Presence of lung point
  ○ Absence of lung sliding
  ○ Absence of lung B lines
  ○ Absence of lung pulse
- **Pneumonia findings**
  ○ Loss of A lines
  ○ Hepatization
  ○ Dynamic air Bronchogram
  ○ Ill-defined Margins
- **COVID 19 Lung Findings**
  ○ Posterior and inferior lung field lesions
  ○ B lines
  ○ Distorted pleural lines
  ○ Subpleural pulmonary consolidation
  ○ Air bronchogram
- **Other POCUS -Pulmonary Findings and Names**
  ○ Bat Sign (Pleural Line) - Periosteum of the ribs represents the wings and the bright hyperechoic pleural line in between them represents the bat’s body.
  ○ Lung rockets – Accumulation of fluid in the pulmonary interstitial space.
  ○ Seashore sign – Demonstrates normal Lung findings under M mode.
  ○ Barcode sign – Demonstrates pneumothorax under M mode.
  ○ Plankton sign – Demonstrates pleural effusion. In large effusions, a lung that has collapsed under the pressure of the fluid collections may be seen sliding in a fluid motion with the patient’s respiratory efforts
  ○ Lung point – highly specific ultrasound sign of pneumothorax. The junction between sliding lung and absent sliding is known as lung point.
  ○ Lung Pulse – rhythmic movement of the pleura in synchrony with the cardiac rhythm. Parietal and visceral pleura oppose one another, and so its presence rules out a pneumothorax.
