# Supplementary material for: Zombie Cruise Ship Virtual Escape Room for POCUS Pulmonary: Scan Your Way Out
Source: J Educ Teach Emerg Med. 2022 Jul 15;7(3):SG1–SG23. doi: 10.21980/J8RM0M (PMC10332700; doi:10.21980/J8RM0M)
Supplement: Supplementary file 1 — PDF file. [file jetem-7-3-sg1-appendixA.pdf]

Zombie Cruise Ship: Scan Your Way Out Instructor Map

<table>
<tr><th colspan="3"><h1>Scan Your Way Out</h1><h2>POCUS Pulmonary</h2></th></tr>
<tr><td>Run Time</td><td colspan="2">~40 minutes</td></tr>
<tr><td>Structure</td><td colspan="2">This activity has two paths:
learners either volunteer to help cruise ship physician or they don’t. There are other “choices” but this first one is what determines their ending.</td></tr>
<tr><td>Tips for Instructors</td><td colspan="2">While learners may not know some of the questions asked, there is no penalty for incorrect answers. It is expected that they will frequently answer questions incorrectly. The format will simply return them to the question to try again, so they should not be able to become stuck.</td></tr>
<tr><td>Tips for Instructors</td><td colspan="2">It is encouraged to allow the learners to struggle on questions and for the instructor to provide elaboration after learners have selected the correct answer.</td></tr>
<tr><td>Link</td><td colspan="2">May give learners link to activity. If being done over Zoom, it is suggested that one learner share the screen and everyone walks through choices as a team.
https://forms.gle/rpykKkH8ayXBCxcb8</td></tr>
<tr><td>Initial</td><td colspan="2">The scene is set that they are enjoying a cruise ship vacation when a mysterious virus spreads and zombies attack starts.</td></tr>
<tr><td>Learning points (in order of appearance)</td><td colspan="2">Learners should be able to perform POCUS pulmonary by selecting an appropriate probe, to identify pulmonary ultrasound pathological findings, and to come up with correct diagnoses and treatment plans.</td></tr>
<tr><td>Choice: Same learning points covered</td><td>Volunteer to help –
Learners will be able to obtain medical supplies, PPE, portable ultrasound and a secret key.</td><td>Nah. I am on a Vacation
Learners will be able to grab a portable ultrasound but no medical supplies, no PPE, and no secret key.</td></tr>
<tr><td>Learning Points (in order of appearance)</td><td colspan="2">Learners recognize an appropriate probe for POCUS lung.</td></tr>
<tr><td></td><td colspan="2">Learners identify signs of absence of lung sliding.</td></tr>
<tr><td></td><td colspan="2">Learners use M mode and are able to identify signs of pneumothorax.</td></tr>
<tr><td></td><td colspan="2">Learners able to recognize cardiac arrest on US.</td></tr>
<tr><td></td><td colspan="2">Learners able to recognize pulmonary edema with increased B lines (more than 3 B lines).</td></tr>
<tr><td></td><td colspan="2">Learners recognize the cause of pulmonary edema is due to heart failure on cardiac ultrasound – decreased left ventricular ejection fraction (LVEF) and contractility.</td></tr>
<tr><td></td><td>Give PO Lasix, obtain a key</td><td>No PO Lasix, NO key</td></tr>
<tr><td></td><td colspan="2">Diagnose pneumonia based on ultrasound findings – dynamic air bronchogram, hepatization, loss of A lines.</td></tr>
</table>

	Learners distinguish difference between atelectasis vs. pneumonia findings on US.
	Learners identify hemothorax on a trauma patient.
	Learners identify pneumothorax on a trauma patient.
	Learners understand ultrasound findings of COVID19 pneumonia.
