# Supplementary material for: Zombie Cruise Ship Virtual Escape Room for POCUS Pulmonary: Scan Your Way Out
Source: J Educ Teach Emerg Med. 2022 Jul 15;7(3):SG1–SG23. doi: 10.21980/J8RM0M (PMC10332700; doi:10.21980/J8RM0M)

## Slide 1
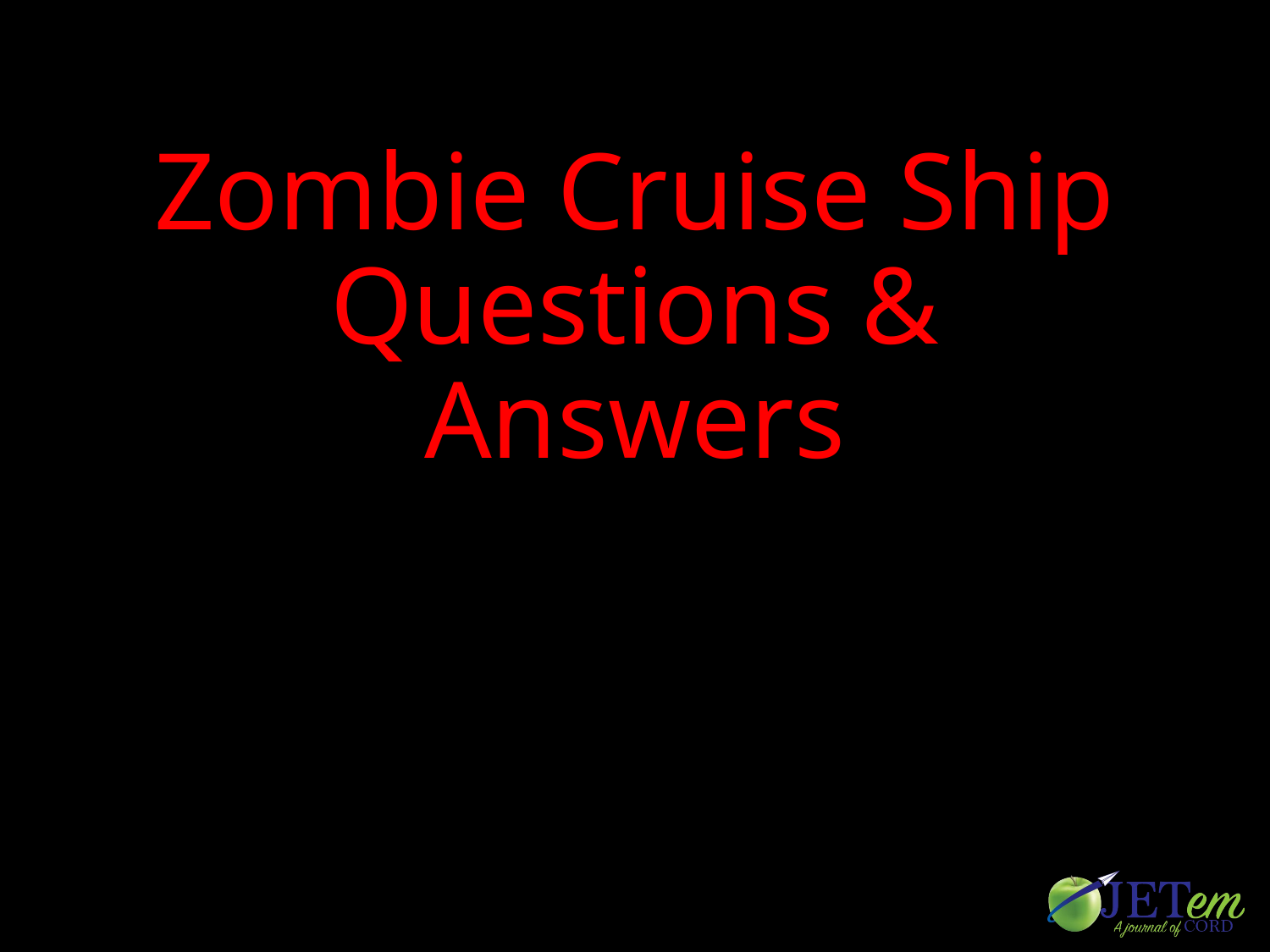

# Zombie Cruise Ship Questions & Answers

## Slide 2
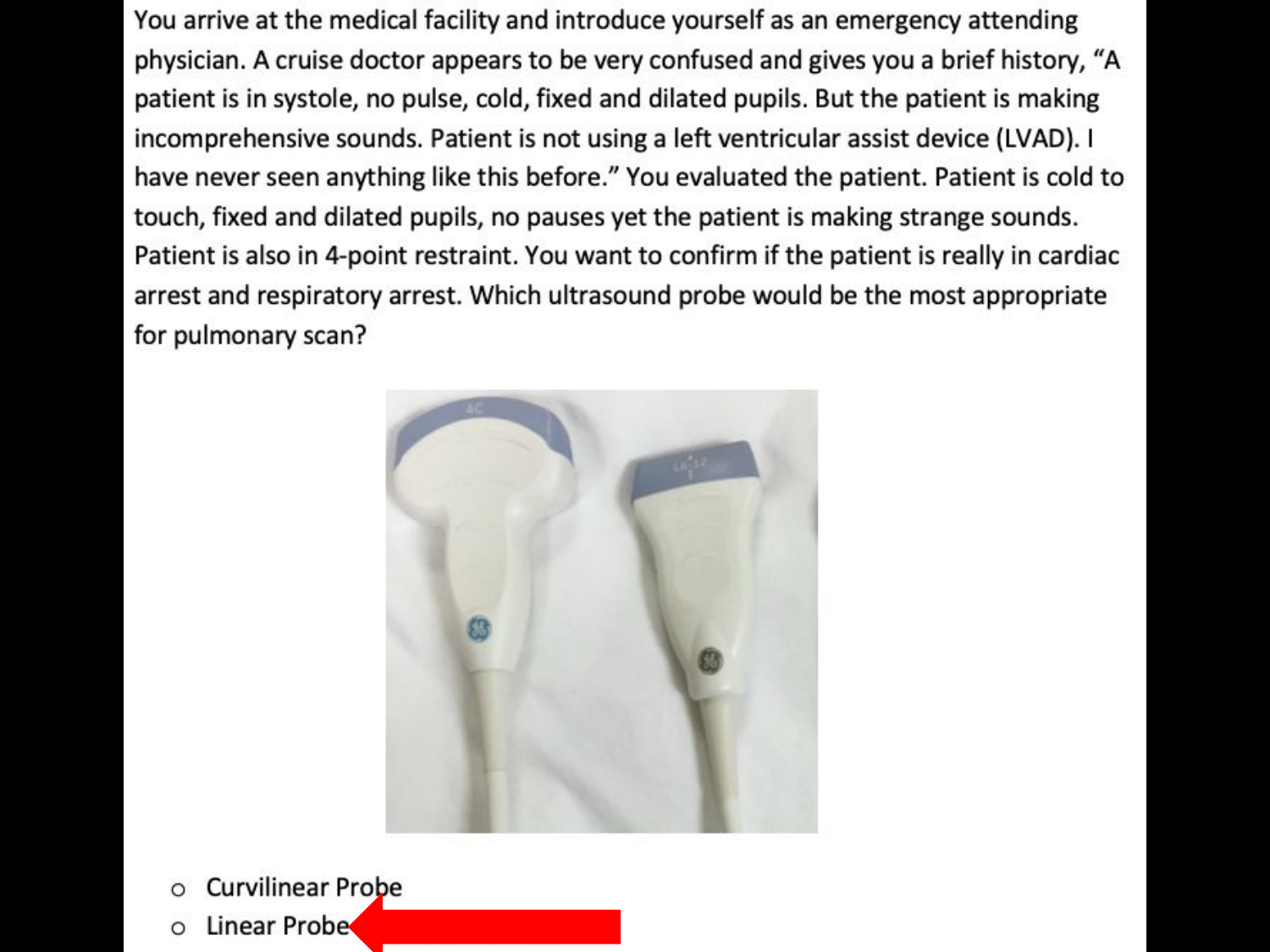

## Slide 3
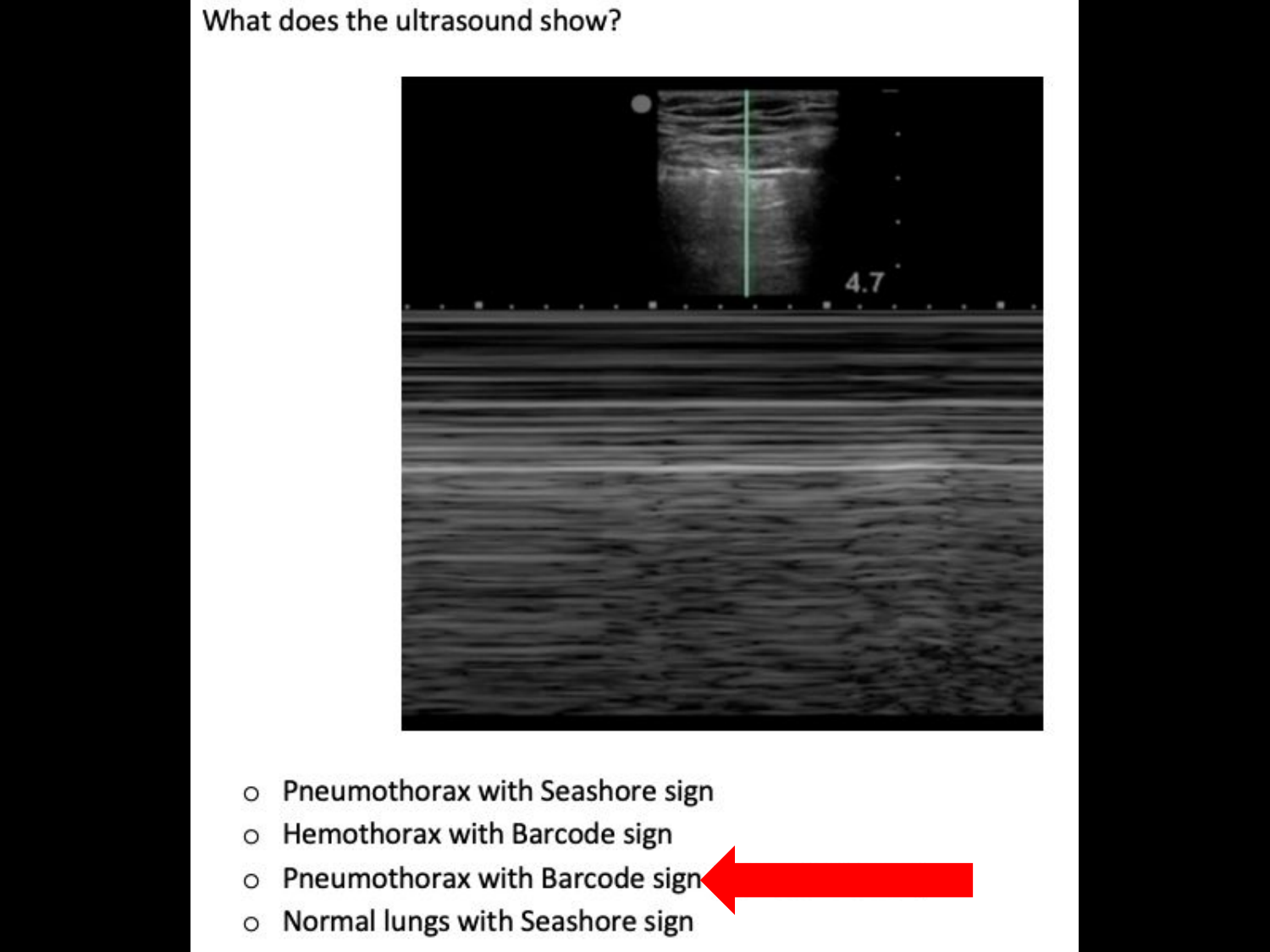

## Slide 4
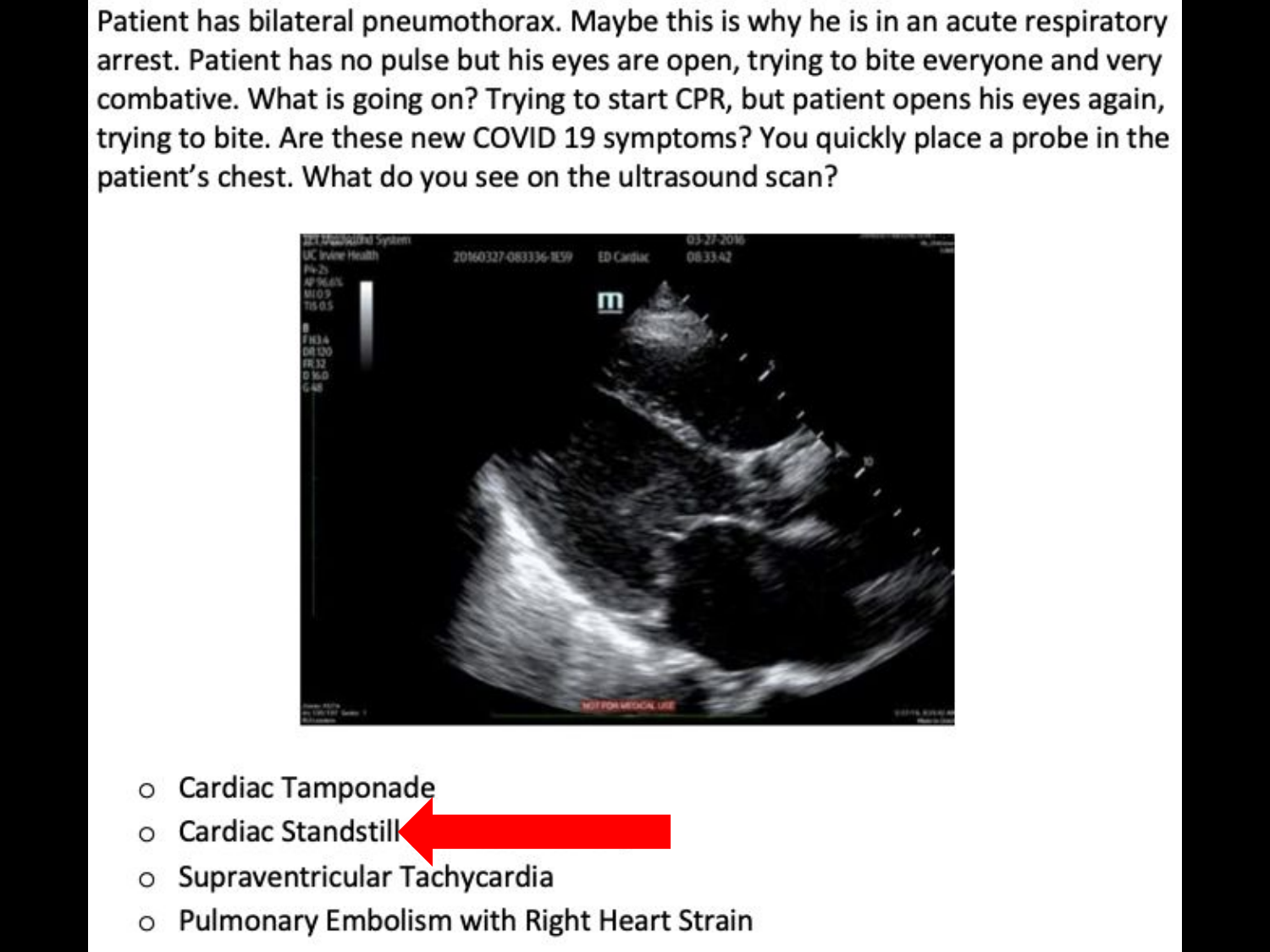

## Slide 5
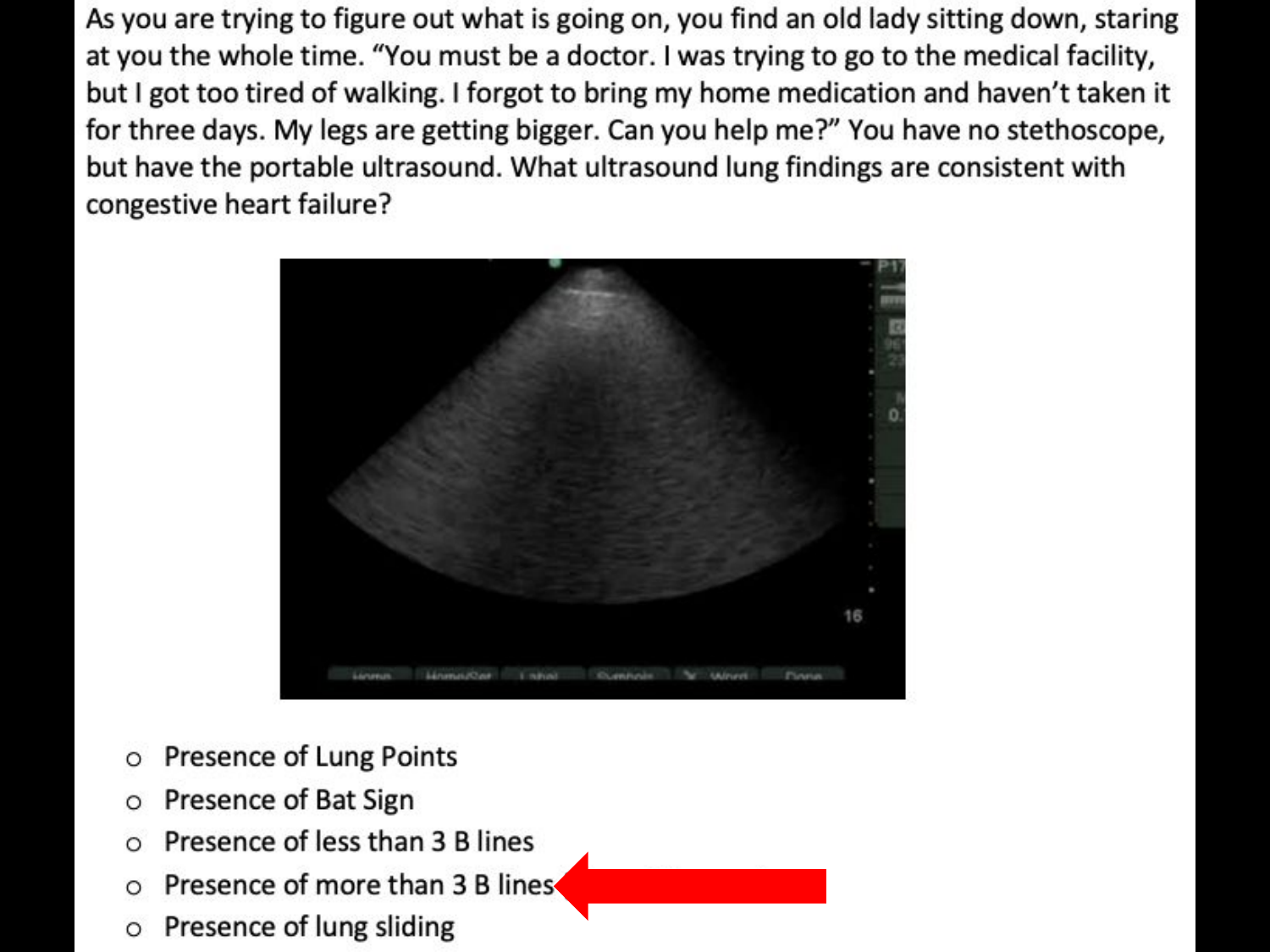

## Slide 6
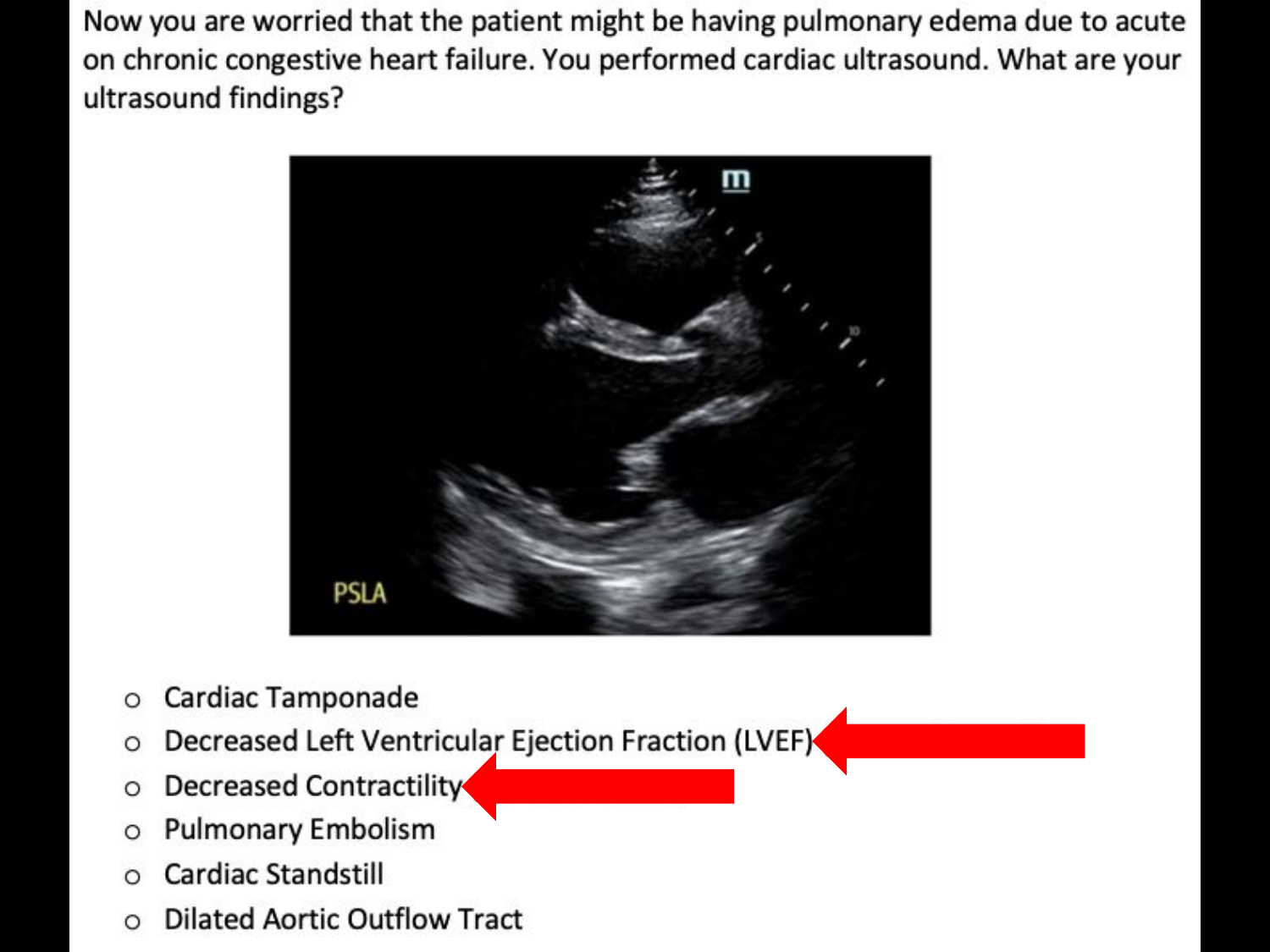

## Slide 7
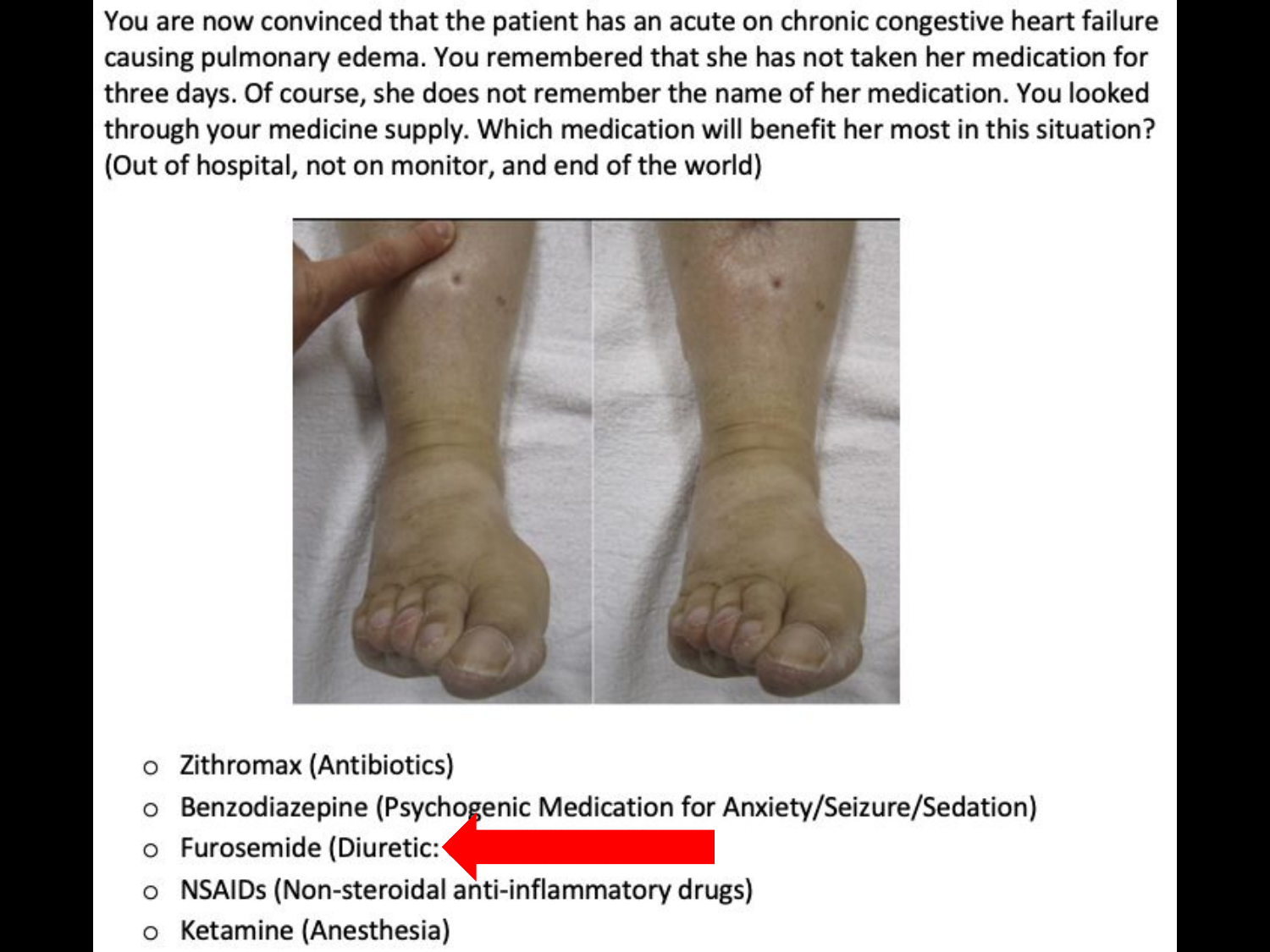

## Slide 8
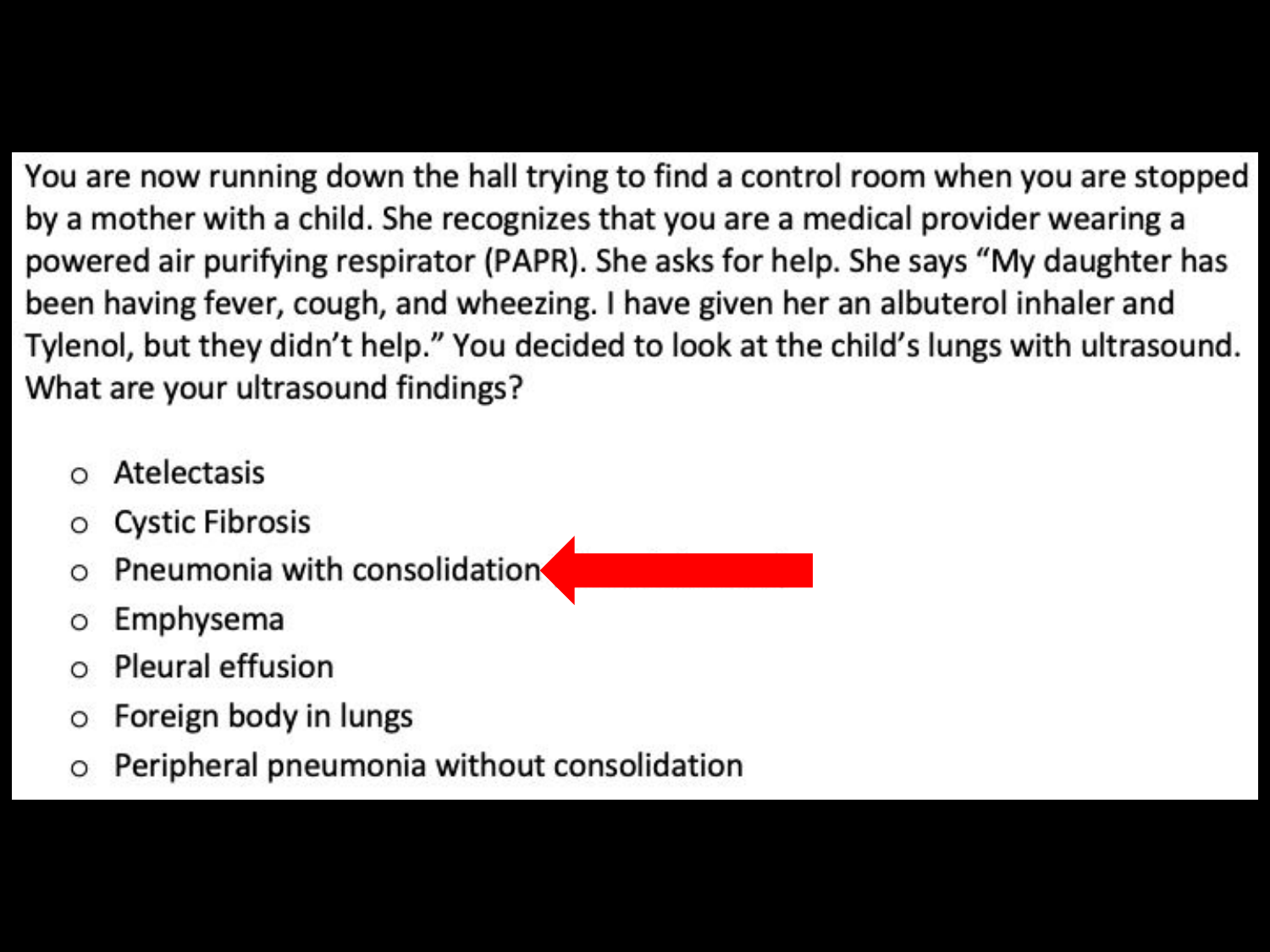

## Slide 9
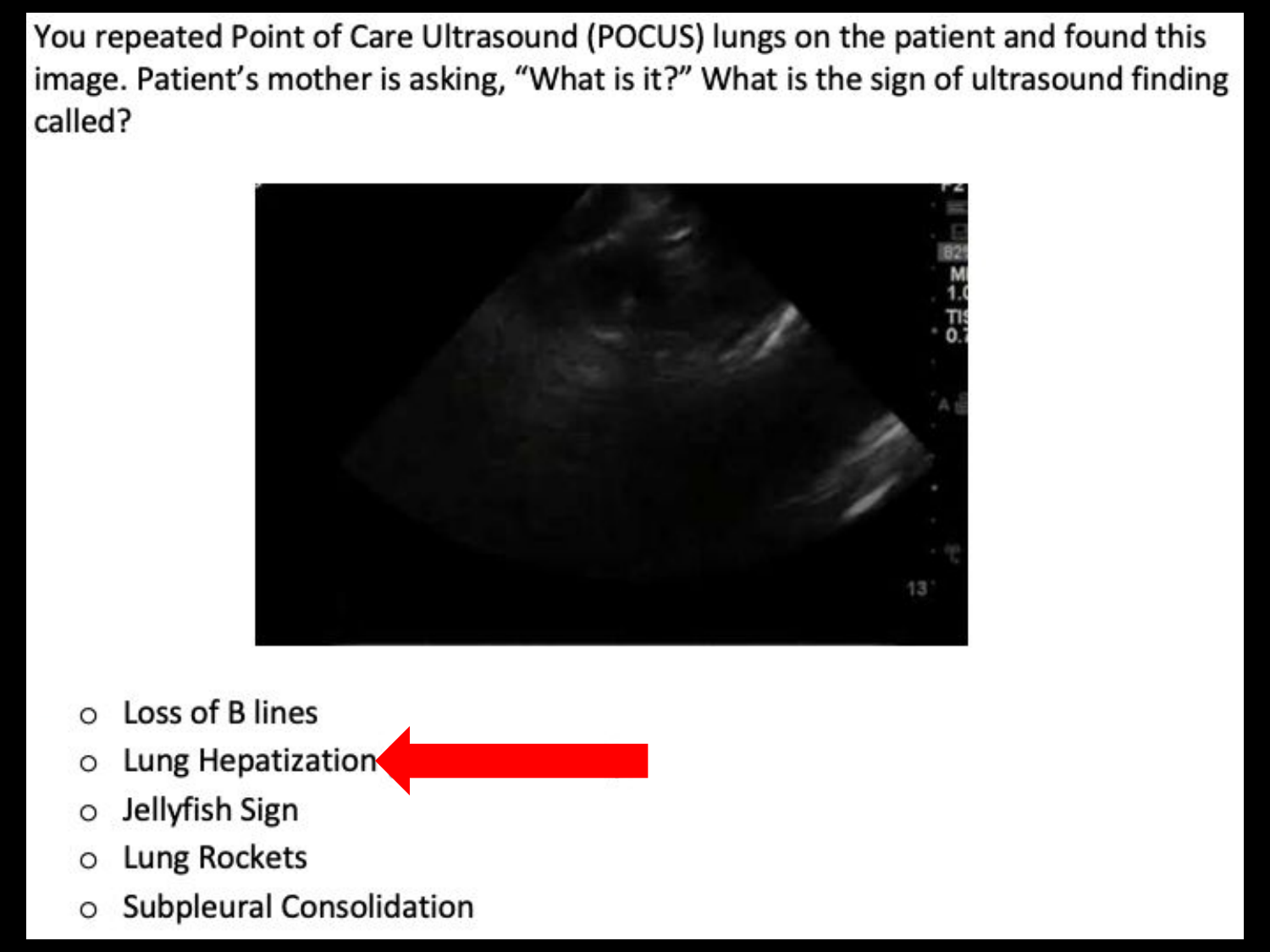

## Slide 10
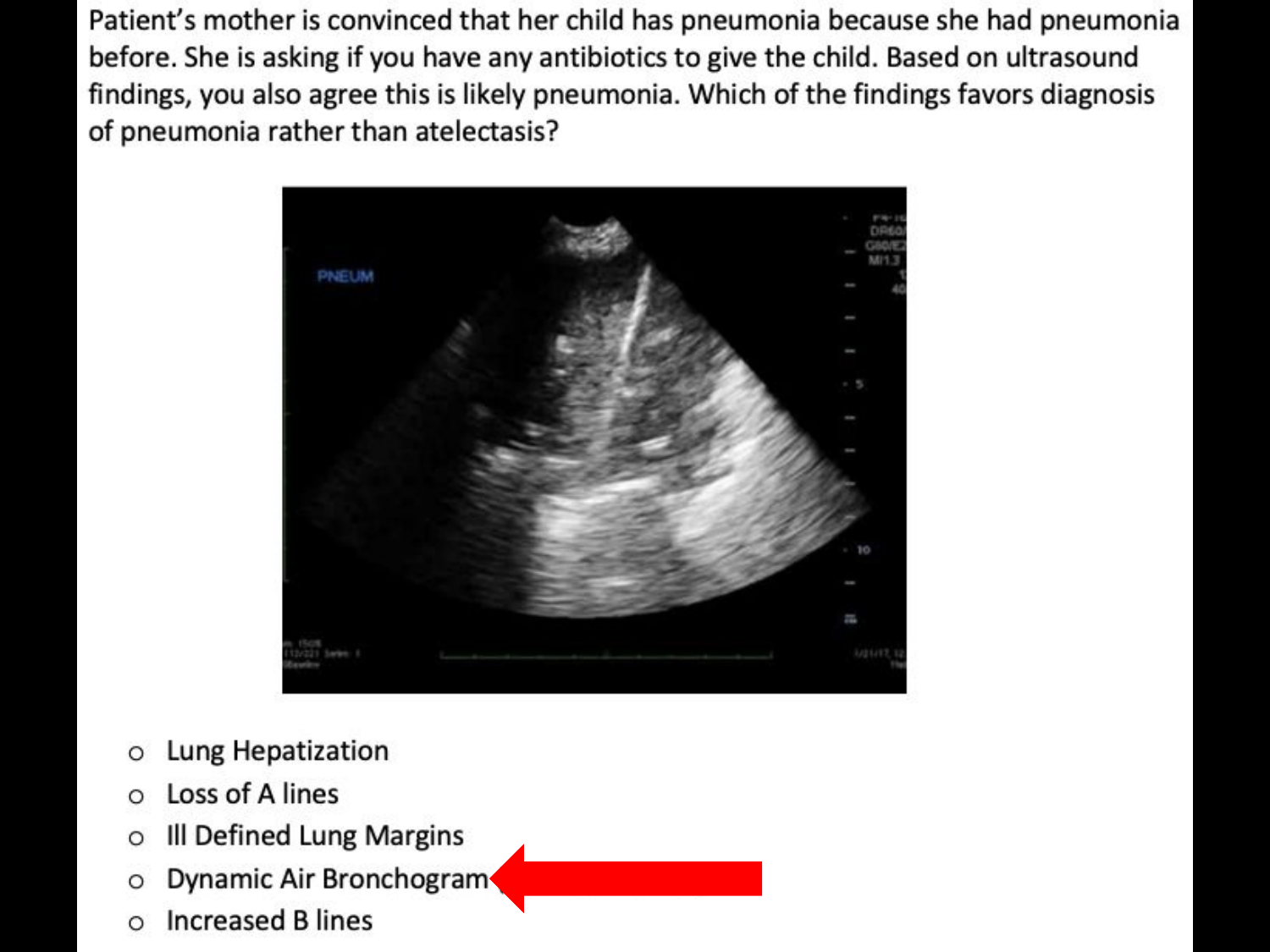

## Slide 11
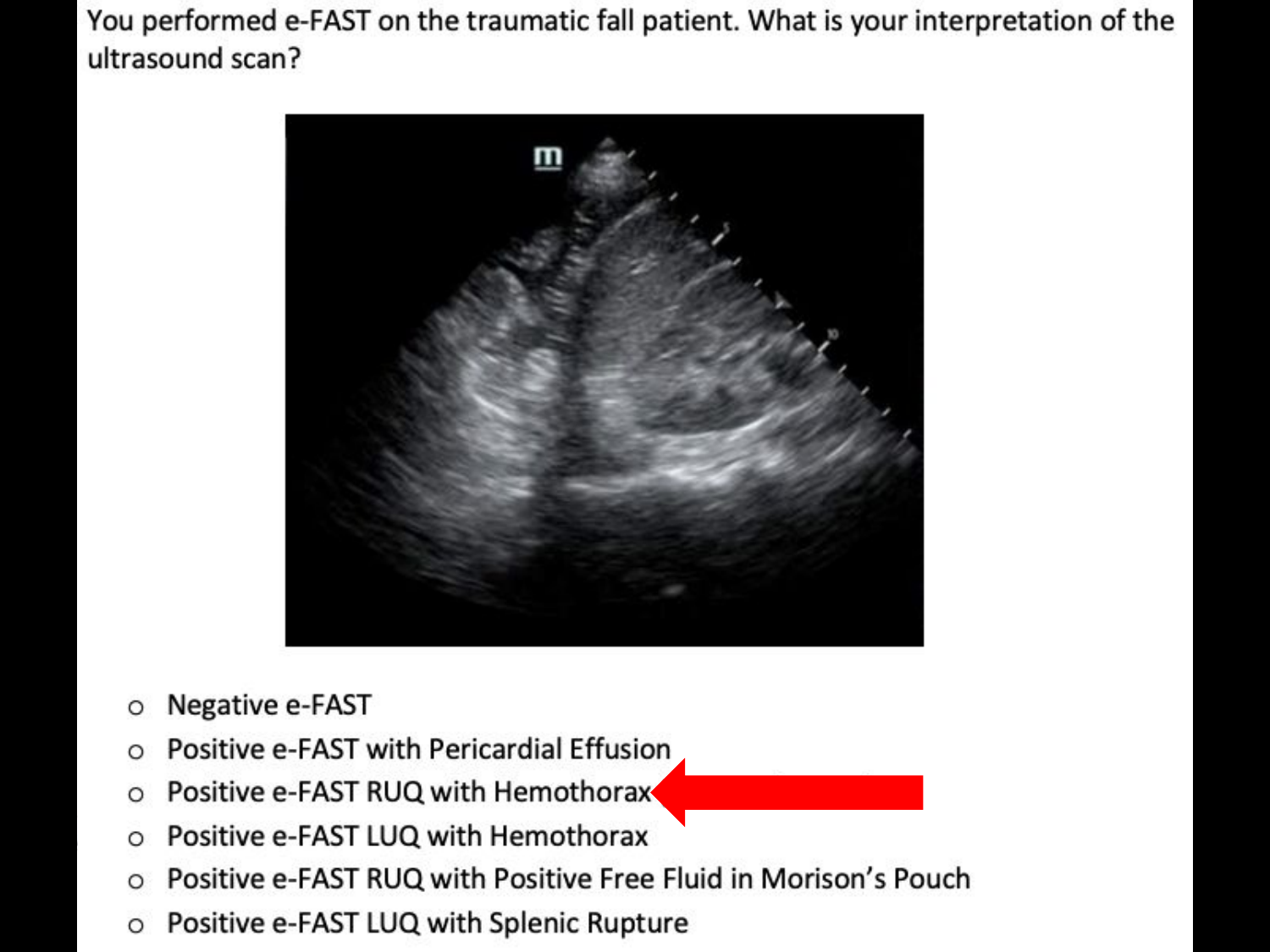

## Slide 12
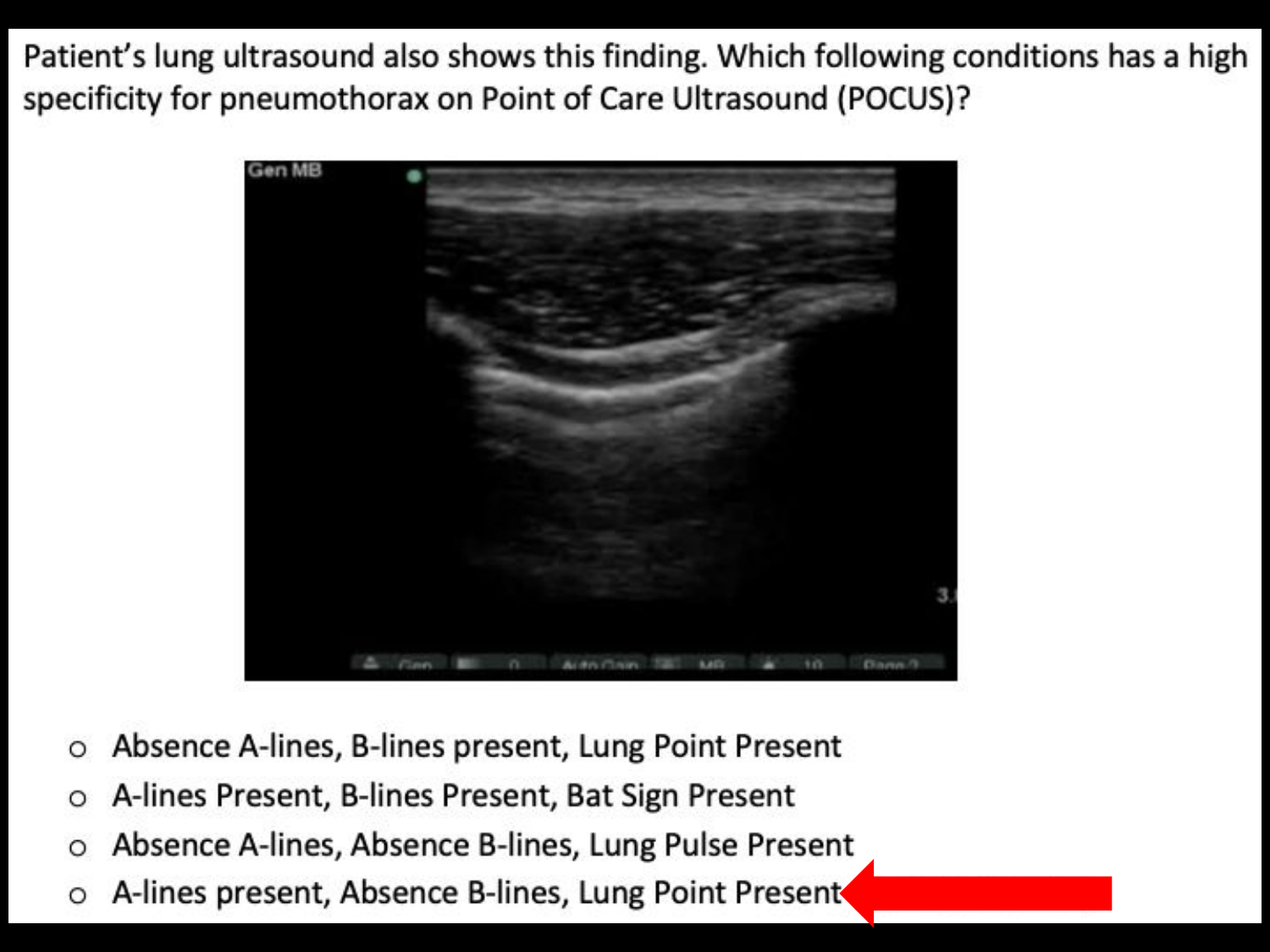

## Slide 13
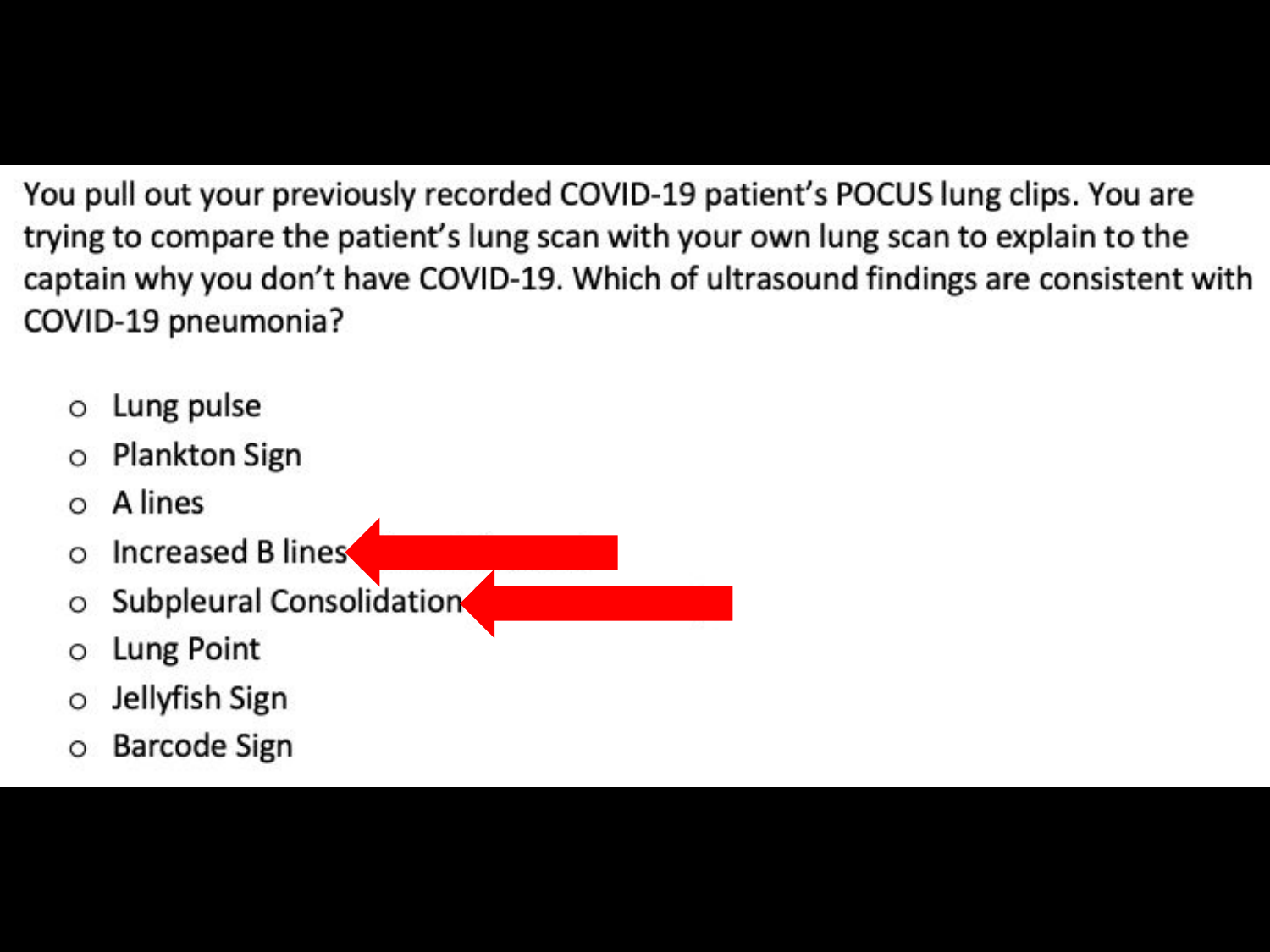

Supplement: Supplementary file 2 — Please see associated PDF file [file jetem-7-3-sg1-appendixE.pptx]
